# Supplementary material for: Resolving the full spectrum of human genome variation using Linked-Reads
Source: Genome Res. 2019 Apr;29(4):635–45. doi: 10.1101/gr.234443.118 (PMC6442396; doi:10.1101/gr.234443.118)
Supplement: Supplemental Material [file supp_gr.234443.118_Supplemental_longranger-2.2.zip › longranger-2.2.tar.bz2!/lr2.2-public/lib/stan/cmdstan/stan/doc/api/html/exp__mod__normal_8hpp_source.html]

Stan: src/stan/prob/distributions/univariate/continuous/exp\_mod\_normal.hpp Source File


|  |  |
| --- | --- |
|  | Stan  2.5.0  probability, sampling & optimization |


- Main Page
- Related Pages
- Namespaces
- Classes
- Files

- File List
- File Members

All Classes Namespaces Files Functions Variables Typedefs Enumerations Enumerator Friends Macros Pages

- src
- stan
- prob
- distributions
- univariate
- continuous

exp\_mod\_normal.hpp

Go to the documentation of this file.

1 #ifndef STAN\_\_PROB\_\_DISTRIBUTIONS\_\_UNIVARIATE\_\_CONTINUOUS\_\_EXP\_\_MOD\_\_NORMAL\_\_HPP

2 #define STAN\_\_PROB\_\_DISTRIBUTIONS\_\_UNIVARIATE\_\_CONTINUOUS\_\_EXP\_\_MOD\_\_NORMAL\_\_HPP

3

4 #include <boost/random/normal\_distribution.hpp>

5 #include <boost/math/special\_functions/fpclassify.hpp>

6 #include <boost/random/variate\_generator.hpp>

7 #include <stan/prob/distributions/univariate/continuous/normal.hpp>

8 #include <stan/prob/distributions/univariate/continuous/exponential.hpp>

9

10 #include <stan/agrad/partials\_vari.hpp>

11 #include <stan/math/error\_handling.hpp>

12 #include <stan/math/constants.hpp>

13 #include <stan/meta/traits.hpp>

14 #include <stan/prob/constants.hpp>

15 #include <stan/prob/traits.hpp>

16 #include <stan/math/functions/value\_of.hpp>

17

18 namespace stan {

19

20  namespace prob {

21

22  template <bool propto,

23  typename T\_y, typename T\_loc, typename T\_scale,

24  typename T\_inv\_scale>

25  typename return\_type<T\_y,T\_loc,T\_scale, T\_inv\_scale>::type

26  exp\_mod\_normal\_log(const T\_y& y, const T\_loc& mu, const T\_scale& sigma,

27  const T\_inv\_scale& lambda) {

28  static const char\* function = "stan::prob::exp\_mod\_normal\_log(%1%)";

29

30  using stan::is\_constant\_struct;

31  using stan::math::check\_positive\_finite;

32  using stan::math::check\_finite;

33  using stan::math::check\_not\_nan;

34  using stan::math::check\_consistent\_sizes;

35  using stan::math::value\_of;

36  using stan::prob::include\_summand;

37

38  // check if any vectors are zero length

39  if (!(stan::length(y)

40  && stan::length(mu)

41  && stan::length(sigma)

42  && stan::length(lambda)))

43  return 0.0;

44

45  // set up return value accumulator

46  double logp(0.0);

47

48  // validate args (here done over var, which should be OK)

49  check\_not\_nan(function, y, "Random variable", &logp);

50  check\_finite(function, mu, "Location parameter", &logp);

51  check\_positive\_finite(function, lambda, "Inv\_scale parameter", &logp);

52  check\_positive\_finite(function, sigma, "Scale parameter", &logp);

53  check\_consistent\_sizes(function,

54  y,mu,sigma,lambda,

55  "Random variable","Location parameter",

56  "Scale parameter", "Inv\_scale paramter",

57  &logp);

58

59  // check if no variables are involved and prop-to

60  if (!include\_summand<propto,T\_y,T\_loc,T\_scale,T\_inv\_scale>::value)

61  return 0.0;

62

63  // set up template expressions wrapping scalars into vector views

64  agrad::OperandsAndPartials<T\_y, T\_loc, T\_scale, T\_inv\_scale>

65  operands\_and\_partials(y, mu, sigma,lambda);

66

67  VectorView<const T\_y> y\_vec(y);

68  VectorView<const T\_loc> mu\_vec(mu);

69  VectorView<const T\_scale> sigma\_vec(sigma);

70  VectorView<const T\_inv\_scale> lambda\_vec(lambda);

71  size\_t N = max\_size(y, mu, sigma, lambda);

72

73  for (size\_t n = 0; n < N; n++) {

74  //pull out values of arguments

75  const double y\_dbl = value\_of(y\_vec[n]);

76  const double mu\_dbl = value\_of(mu\_vec[n]);

77  const double sigma\_dbl = value\_of(sigma\_vec[n]);

78  const double lambda\_dbl = value\_of(lambda\_vec[n]);

79

80  const double pi\_dbl = boost::math::constants::pi<double>();

81

82  // log probability

83  if (include\_summand<propto>::value)

84  logp -= log(2.0);

85  if (include\_summand<propto, T\_inv\_scale>::value)

86  logp += log(lambda\_dbl);

87  if (include\_summand<propto,T\_y,T\_loc,T\_scale,T\_inv\_scale>::value)

88  logp += lambda\_dbl

89  \* (mu\_dbl + 0.5 \* lambda\_dbl \* sigma\_dbl \* sigma\_dbl - y\_dbl)

90  + log(boost::math::erfc((mu\_dbl + lambda\_dbl \* sigma\_dbl

91  \* sigma\_dbl - y\_dbl)

92  / (std::sqrt(2.0) \* sigma\_dbl)));

93

94  // gradients

95  const double deriv\_logerfc

96  = -2.0 / std::sqrt(pi\_dbl)

97  \* exp(-(mu\_dbl + lambda\_dbl \* sigma\_dbl \* sigma\_dbl - y\_dbl)

98  / (std::sqrt(2.0) \* sigma\_dbl)

99  \* (mu\_dbl + lambda\_dbl \* sigma\_dbl \* sigma\_dbl - y\_dbl)

100  / (sigma\_dbl \* std::sqrt(2.0)))

101  / boost::math::erfc((mu\_dbl + lambda\_dbl \* sigma\_dbl \* sigma\_dbl

102  - y\_dbl)

103  / (sigma\_dbl \* std::sqrt(2.0)));

104

105  if (!is\_constant\_struct<T\_y>::value)

106  operands\_and\_partials.d\_x1[n]

107  += -lambda\_dbl

108  + deriv\_logerfc \* -1.0 / (sigma\_dbl \* std::sqrt(2.0));

109  if (!is\_constant\_struct<T\_loc>::value)

110  operands\_and\_partials.d\_x2[n]

111  += lambda\_dbl

112  + deriv\_logerfc / (sigma\_dbl \* std::sqrt(2.0));

113  if (!is\_constant\_struct<T\_scale>::value)

114  operands\_and\_partials.d\_x3[n]

115  += sigma\_dbl \* lambda\_dbl \* lambda\_dbl

116  + deriv\_logerfc

117  \* (-mu\_dbl / (sigma\_dbl \* sigma\_dbl \* std::sqrt(2.0))

118  + lambda\_dbl / std::sqrt(2.0)

119  + y\_dbl / (sigma\_dbl \* sigma\_dbl \* std::sqrt(2.0)));

120  if (!is\_constant\_struct<T\_inv\_scale>::value)

121  operands\_and\_partials.d\_x4[n]

122  += 1 / lambda\_dbl + lambda\_dbl \* sigma\_dbl \* sigma\_dbl

123  + mu\_dbl - y\_dbl + deriv\_logerfc \* sigma\_dbl / std::sqrt(2.0);

124  }

125  return operands\_and\_partials.to\_var(logp);

126  }

127

128  template <typename T\_y, typename T\_loc, typename T\_scale,

129  typename T\_inv\_scale>

130  inline

131  typename return\_type<T\_y,T\_loc,T\_scale, T\_inv\_scale>::type

132  exp\_mod\_normal\_log(const T\_y& y, const T\_loc& mu, const T\_scale& sigma,

133  const T\_inv\_scale& lambda) {

134  return exp\_mod\_normal\_log<false>(y,mu,sigma,lambda);

135  }

136

137  template <typename T\_y, typename T\_loc, typename T\_scale,

138  typename T\_inv\_scale>

139  typename return\_type<T\_y,T\_loc,T\_scale,T\_inv\_scale>::type

140  exp\_mod\_normal\_cdf(const T\_y& y, const T\_loc& mu, const T\_scale& sigma,

141  const T\_inv\_scale& lambda) {

142  static const char\* function = "stan::prob::exp\_mod\_normal\_cdf(%1%)";

143

144  using stan::math::check\_positive\_finite;

145  using stan::math::check\_finite;

146  using stan::math::check\_not\_nan;

147  using stan::math::check\_consistent\_sizes;

148  using stan::math::value\_of;

149

150  double cdf(1.0);

151  //check if any vectors are zero length

152  if (!(stan::length(y)

153  && stan::length(mu)

154  && stan::length(sigma)

155  && stan::length(lambda)))

156  return cdf;

157

158  check\_not\_nan(function, y, "Random variable", &cdf);

159  check\_finite(function, mu, "Location parameter", &cdf);

160  check\_not\_nan(function, sigma, "Scale parameter", &cdf);

161  check\_positive\_finite(function, sigma, "Scale parameter", &cdf);

162  check\_positive\_finite(function, lambda, "Inv\_scale parameter", &cdf);

163  check\_not\_nan(function, lambda, "Inv\_scale parameter", &cdf);

164  check\_consistent\_sizes(function,

165  y,mu,sigma,lambda,

166  "Random variable","Location parameter",

167  "Scale parameter","Inv\_scale paramter",

168  &cdf);

169

170  agrad::OperandsAndPartials<T\_y, T\_loc, T\_scale, T\_inv\_scale>

171  operands\_and\_partials(y, mu, sigma,lambda);

172

173  using stan::math::SQRT\_2;

174

175  VectorView<const T\_y> y\_vec(y);

176  VectorView<const T\_loc> mu\_vec(mu);

177  VectorView<const T\_scale> sigma\_vec(sigma);

178  VectorView<const T\_inv\_scale> lambda\_vec(lambda);

179  size\_t N = max\_size(y, mu, sigma, lambda);

180  const double sqrt\_pi = std::sqrt(stan::math::pi());

181  for (size\_t n = 0; n < N; n++) {

182

183  if(boost::math::isinf(y\_vec[n])) {

184  if (y\_vec[n] < 0.0)

185  return operands\_and\_partials.to\_var(0.0);

186  }

187

188  const double y\_dbl = value\_of(y\_vec[n]);

189  const double mu\_dbl = value\_of(mu\_vec[n]);

190  const double sigma\_dbl = value\_of(sigma\_vec[n]);

191  const double lambda\_dbl = value\_of(lambda\_vec[n]);

192  const double u = lambda\_dbl \* (y\_dbl - mu\_dbl);

193  const double v = lambda\_dbl \* sigma\_dbl ;

194  const double v\_sq = v \* v;

195  const double scaled\_diff = (y\_dbl - mu\_dbl) / (SQRT\_2 \* sigma\_dbl);

196  const double scaled\_diff\_sq = scaled\_diff \* scaled\_diff;

197  const double erf\_calc = 0.5 \* (1 + erf(-v / SQRT\_2 + scaled\_diff));

198  const double deriv\_1 = lambda\_dbl \* exp(0.5 \* v\_sq - u) \* erf\_calc;

199  const double deriv\_2 = SQRT\_2 / sqrt\_pi \* 0.5 \* exp(0.5 \* v\_sq

200  - (scaled\_diff

201  - (v / SQRT\_2)) \* (scaled\_diff

202  - (v / SQRT\_2)) - u) / sigma\_dbl;

203  const double deriv\_3 = SQRT\_2 / sqrt\_pi \* 0.5 \* exp(-scaled\_diff\_sq)

204  / sigma\_dbl;

205

206  const double cdf\_ = 0.5 \* (1 + erf(u / (v \* SQRT\_2)))

207  - exp(-u + v\_sq \* 0.5) \* (erf\_calc);

208

209  cdf \*= cdf\_;

210

211  if (!is\_constant\_struct<T\_y>::value)

212  operands\_and\_partials.d\_x1[n] += (deriv\_1 - deriv\_2 + deriv\_3)

213  / cdf\_;

214  if (!is\_constant\_struct<T\_loc>::value)

215  operands\_and\_partials.d\_x2[n] += (-deriv\_1 + deriv\_2 - deriv\_3)

216  / cdf\_;

217  if (!is\_constant\_struct<T\_scale>::value)

218  operands\_and\_partials.d\_x3[n] += (-deriv\_1 \* v - deriv\_3

219  \* scaled\_diff \* SQRT\_2 - deriv\_2 \* sigma\_dbl \* SQRT\_2 \* (-SQRT\_2

220  \* 0.5 \* (-lambda\_dbl + scaled\_diff \* SQRT\_2 / sigma\_dbl) - SQRT\_2

221  \* lambda\_dbl)) / cdf\_;

222  if (!is\_constant\_struct<T\_inv\_scale>::value)

223  operands\_and\_partials.d\_x4[n] += exp(0.5 \* v\_sq - u) \* (SQRT\_2

224  / sqrt\_pi \* 0.5 \* sigma\_dbl \* exp(-(v / SQRT\_2 - scaled\_diff) \* (v

225  / SQRT\_2 - scaled\_diff)) - (v \* sigma\_dbl + mu\_dbl - y\_dbl)

226  \* erf\_calc) / cdf\_;

227  }

228

229  if (!is\_constant\_struct<T\_y>::value) {

230  for(size\_t n = 0; n < stan::length(y); ++n)

231  operands\_and\_partials.d\_x1[n] \*= cdf;

232  }

233  if (!is\_constant\_struct<T\_loc>::value) {

234  for(size\_t n = 0; n < stan::length(mu); ++n)

235  operands\_and\_partials.d\_x2[n] \*= cdf;

236  }

237  if (!is\_constant\_struct<T\_scale>::value) {

238  for(size\_t n = 0; n < stan::length(sigma); ++n)

239  operands\_and\_partials.d\_x3[n] \*= cdf;

240  }

241  if (!is\_constant\_struct<T\_inv\_scale>::value) {

242  for(size\_t n = 0; n < stan::length(lambda); ++n)

243  operands\_and\_partials.d\_x4[n] \*= cdf;

244  }

245

246  return operands\_and\_partials.to\_var(cdf);

247  }

248

249  template <typename T\_y, typename T\_loc, typename T\_scale,

250  typename T\_inv\_scale>

251  typename return\_type<T\_y,T\_loc,T\_scale,T\_inv\_scale>::type

252  exp\_mod\_normal\_cdf\_log(const T\_y& y, const T\_loc& mu, const T\_scale& sigma,

253  const T\_inv\_scale& lambda) {

254  static const char\* function = "stan::prob::exp\_mod\_normal\_cdf\_log(%1%)";

255

256  using stan::math::check\_positive\_finite;

257  using stan::math::check\_finite;

258  using stan::math::check\_not\_nan;

259  using stan::math::check\_consistent\_sizes;

260  using stan::math::value\_of;

261

262  double cdf\_log(0.0);

263  //check if any vectors are zero length

264  if (!(stan::length(y)

265  && stan::length(mu)

266  && stan::length(sigma)

267  && stan::length(lambda)))

268  return cdf\_log;

269

270  check\_not\_nan(function, y, "Random variable", &cdf\_log);

271  check\_finite(function, mu, "Location parameter", &cdf\_log);

272  check\_not\_nan(function, sigma, "Scale parameter", &cdf\_log);

273  check\_positive\_finite(function, sigma, "Scale parameter", &cdf\_log);

274  check\_positive\_finite(function, lambda, "Inv\_scale parameter", &cdf\_log);

275  check\_not\_nan(function, lambda, "Inv\_scale parameter", &cdf\_log);

276  check\_consistent\_sizes(function,

277  y,mu,sigma,lambda,

278  "Random variable","Location parameter",

279  "Scale parameter","Inv\_scale paramter",

280  &cdf\_log);

281

282  agrad::OperandsAndPartials<T\_y, T\_loc, T\_scale, T\_inv\_scale>

283  operands\_and\_partials(y, mu, sigma,lambda);

284

285  using stan::math::SQRT\_2;

286  using std::log;

287

288  VectorView<const T\_y> y\_vec(y);

289  VectorView<const T\_loc> mu\_vec(mu);

290  VectorView<const T\_scale> sigma\_vec(sigma);

291  VectorView<const T\_inv\_scale> lambda\_vec(lambda);

292  size\_t N = max\_size(y, mu, sigma, lambda);

293  const double sqrt\_pi = std::sqrt(stan::math::pi());

294  for (size\_t n = 0; n < N; n++) {

295

296  if(boost::math::isinf(y\_vec[n])) {

297  if (y\_vec[n] < 0.0)

298  return operands\_and\_partials.to\_var(stan::math::negative\_infinity());

299  else

300  return operands\_and\_partials.to\_var(0.0);

301  }

302

303  const double y\_dbl = value\_of(y\_vec[n]);

304  const double mu\_dbl = value\_of(mu\_vec[n]);

305  const double sigma\_dbl = value\_of(sigma\_vec[n]);

306  const double lambda\_dbl = value\_of(lambda\_vec[n]);

307  const double u = lambda\_dbl \* (y\_dbl - mu\_dbl);

308  const double v = lambda\_dbl \* sigma\_dbl ;

309  const double v\_sq = v \* v;

310  const double scaled\_diff = (y\_dbl - mu\_dbl) / (SQRT\_2 \* sigma\_dbl);

311  const double scaled\_diff\_sq = scaled\_diff \* scaled\_diff;

312  const double erf\_calc1 = 0.5 \* (1 + erf(u / (v \* SQRT\_2)));

313  const double erf\_calc2 = 0.5 \* (1 + erf(u / (v \* SQRT\_2) - v / SQRT\_2));

314  const double deriv\_1 = lambda\_dbl \* exp(0.5 \* v\_sq - u) \* erf\_calc2;

315  const double deriv\_2 = SQRT\_2 / sqrt\_pi \* 0.5

316  \* exp(0.5 \* v\_sq - (-scaled\_diff + (v / SQRT\_2))

317  \* (-scaled\_diff + (v / SQRT\_2)) - u) / sigma\_dbl;

318  const double deriv\_3 = SQRT\_2 / sqrt\_pi \* 0.5 \* exp(-scaled\_diff\_sq)

319  / sigma\_dbl;

320

321  const double denom = erf\_calc1 - erf\_calc2 \* exp(0.5 \* v\_sq - u);

322  const double cdf\_ = erf\_calc1 - exp(-u + v\_sq \* 0.5) \* (erf\_calc2);

323

324  cdf\_log += log(cdf\_);

325

326  if (!is\_constant\_struct<T\_y>::value)

327  operands\_and\_partials.d\_x1[n] += (deriv\_1 - deriv\_2 + deriv\_3)

328  / denom;

329  if (!is\_constant\_struct<T\_loc>::value)

330  operands\_and\_partials.d\_x2[n] += (-deriv\_1 + deriv\_2 - deriv\_3)

331  / denom;

332  if (!is\_constant\_struct<T\_scale>::value)

333  operands\_and\_partials.d\_x3[n]

334  += (-deriv\_1 \* v - deriv\_3 \* scaled\_diff

335  \* SQRT\_2 - deriv\_2 \* sigma\_dbl \* SQRT\_2

336  \* (-SQRT\_2 \* 0.5 \* (-lambda\_dbl + scaled\_diff \* SQRT\_2

337  / sigma\_dbl)

338  - SQRT\_2 \* lambda\_dbl))

339  / denom;

340  if (!is\_constant\_struct<T\_inv\_scale>::value)

341  operands\_and\_partials.d\_x4[n]

342  += exp(0.5 \* v\_sq - u)

343  \* (SQRT\_2 / sqrt\_pi \* 0.5 \* sigma\_dbl

344  \* exp(-(v / SQRT\_2 - scaled\_diff)

345  \* (v / SQRT\_2 - scaled\_diff))

346  - (v \* sigma\_dbl + mu\_dbl - y\_dbl) \* erf\_calc2)

347  / denom;

348  }

349

350  return operands\_and\_partials.to\_var(cdf\_log);

351  }

352

353  template <typename T\_y, typename T\_loc, typename T\_scale,

354  typename T\_inv\_scale>

355  typename return\_type<T\_y,T\_loc,T\_scale,T\_inv\_scale>::type

356  exp\_mod\_normal\_ccdf\_log(const T\_y& y, const T\_loc& mu, const T\_scale& sigma,

357  const T\_inv\_scale& lambda) {

358  static const char\* function = "stan::prob::exp\_mod\_normal\_ccdf\_log(%1%)";

359

360  using stan::math::check\_positive\_finite;

361  using stan::math::check\_finite;

362  using stan::math::check\_not\_nan;

363  using stan::math::check\_consistent\_sizes;

364  using stan::math::value\_of;

365

366  double ccdf\_log(0.0);

367  //check if any vectors are zero length

368  if (!(stan::length(y)

369  && stan::length(mu)

370  && stan::length(sigma)

371  && stan::length(lambda)))

372  return ccdf\_log;

373

374  check\_not\_nan(function, y, "Random variable", &ccdf\_log);

375  check\_finite(function, mu, "Location parameter", &ccdf\_log);

376  check\_not\_nan(function, sigma, "Scale parameter", &ccdf\_log);

377  check\_positive\_finite(function, sigma, "Scale parameter", &ccdf\_log);

378  check\_positive\_finite(function, lambda, "Inv\_scale parameter", &ccdf\_log);

379  check\_not\_nan(function, lambda, "Inv\_scale parameter", &ccdf\_log);

380  check\_consistent\_sizes(function,

381  y,mu,sigma,lambda,

382  "Random variable","Location parameter",

383  "Scale parameter","Inv\_scale paramter",

384  &ccdf\_log);

385

386  agrad::OperandsAndPartials<T\_y, T\_loc, T\_scale, T\_inv\_scale>

387  operands\_and\_partials(y, mu, sigma,lambda);

388

389  using stan::math::SQRT\_2;

390  using std::log;

391

392  VectorView<const T\_y> y\_vec(y);

393  VectorView<const T\_loc> mu\_vec(mu);

394  VectorView<const T\_scale> sigma\_vec(sigma);

395  VectorView<const T\_inv\_scale> lambda\_vec(lambda);

396  size\_t N = max\_size(y, mu, sigma, lambda);

397  const double sqrt\_pi = std::sqrt(stan::math::pi());

398  for (size\_t n = 0; n < N; n++) {

399

400  if(boost::math::isinf(y\_vec[n])) {

401  if (y\_vec[n] > 0.0)

402  return operands\_and\_partials.to\_var(stan::math::negative\_infinity());

403  else

404  return operands\_and\_partials.to\_var(0.0);

405  }

406

407  const double y\_dbl = value\_of(y\_vec[n]);

408  const double mu\_dbl = value\_of(mu\_vec[n]);

409  const double sigma\_dbl = value\_of(sigma\_vec[n]);

410  const double lambda\_dbl = value\_of(lambda\_vec[n]);

411  const double u = lambda\_dbl \* (y\_dbl - mu\_dbl);

412  const double v = lambda\_dbl \* sigma\_dbl ;

413  const double v\_sq = v \* v;

414  const double scaled\_diff = (y\_dbl - mu\_dbl) / (SQRT\_2 \* sigma\_dbl);

415  const double scaled\_diff\_sq = scaled\_diff \* scaled\_diff;

416  const double erf\_calc1 = 0.5 \* (1 + erf(u / (v \* SQRT\_2)));

417  const double erf\_calc2 = 0.5 \* (1 + erf(u / (v \* SQRT\_2) - v / SQRT\_2));

418

419  const double deriv\_1 = lambda\_dbl \* exp(0.5 \* v\_sq - u) \* erf\_calc2;

420  const double deriv\_2 = SQRT\_2 / sqrt\_pi \* 0.5

421  \* exp(0.5 \* v\_sq

422  - (-scaled\_diff + (v / SQRT\_2)) \* (-scaled\_diff

423  + (v / SQRT\_2)) - u)

424  / sigma\_dbl;

425  const double deriv\_3 = SQRT\_2 / sqrt\_pi \* 0.5 \* exp(-scaled\_diff\_sq) / sigma\_dbl;

426

427  const double ccdf\_ = 1.0 - erf\_calc1 + exp(-u + v\_sq \* 0.5) \* (erf\_calc2);

428

429  ccdf\_log += log(ccdf\_);

430

431  if (!is\_constant\_struct<T\_y>::value)

432  operands\_and\_partials.d\_x1[n]

433  -= (deriv\_1 - deriv\_2 + deriv\_3) / ccdf\_;

434  if (!is\_constant\_struct<T\_loc>::value)

435  operands\_and\_partials.d\_x2[n]

436  -= (-deriv\_1 + deriv\_2 - deriv\_3) / ccdf\_;

437  if (!is\_constant\_struct<T\_scale>::value)

438  operands\_and\_partials.d\_x3[n]

439  -= (-deriv\_1 \* v - deriv\_3 \* scaled\_diff \* SQRT\_2 - deriv\_2

440  \* sigma\_dbl \* SQRT\_2

441  \* (-SQRT\_2 \* 0.5 \* (-lambda\_dbl + scaled\_diff \* SQRT\_2

442  / sigma\_dbl)

443  - SQRT\_2 \* lambda\_dbl))

444  / ccdf\_;

445  if (!is\_constant\_struct<T\_inv\_scale>::value)

446  operands\_and\_partials.d\_x4[n] -= exp(0.5 \* v\_sq - u)

447  \* (SQRT\_2 / sqrt\_pi \* 0.5 \* sigma\_dbl

448  \* exp(-(v / SQRT\_2 - scaled\_diff) \* (v / SQRT\_2 - scaled\_diff))

449  - (v \* sigma\_dbl + mu\_dbl - y\_dbl) \* erf\_calc2)

450  / ccdf\_;

451  }

452

453  return operands\_and\_partials.to\_var(ccdf\_log);

454  }

455

456  template <class RNG>

457  inline double

458  exp\_mod\_normal\_rng(const double mu,

459  const double sigma,

460  const double lambda,

461  RNG& rng) {

462

463  static const char\* function = "stan::prob::exp\_mod\_normal\_rng(%1%)";

464

465  using stan::math::check\_positive\_finite;

466  using stan::math::check\_finite;

467

468  check\_finite(function, mu, "Location parameter", (double\*)0);

469  check\_positive\_finite(function, lambda, "Inv\_scale parameter",

470  (double\*)0);

471  check\_positive\_finite(function, sigma, "Scale parameter", (double\*)0);

472

473  return stan::prob::normal\_rng(mu, sigma,rng) + stan::prob::exponential\_rng(lambda, rng);

474  }

475  }

476 }

477 #endif

478

479

480

stan::prob::exp\_mod\_normal\_cdf

return\_type< T\_y, T\_loc, T\_scale, T\_inv\_scale >::type exp\_mod\_normal\_cdf(const T\_y &y, const T\_loc &mu, const T\_scale &sigma, const T\_inv\_scale &lambda)

**Definition:** exp\_mod\_normal.hpp:140

traits.hpp

stan::agrad::erf

fvar< T > erf(const fvar< T > &x)

**Definition:** erf.hpp:17

stan::agrad::OperandsAndPartials::to\_var

T\_return\_type to\_var(double logp)

**Definition:** partials\_vari.hpp:138

stan::math::check\_positive\_finite

bool check\_positive\_finite(const char \*function, const T\_y &y, const char \*name, T\_result \*result)

**Definition:** check\_positive\_finite.hpp:12

error\_handling.hpp

stan::length

size\_t length(const T &)

**Definition:** traits.hpp:159

stan::agrad::erfc

fvar< T > erfc(const fvar< T > &x)

**Definition:** erfc.hpp:17

stan::math::check\_finite

bool check\_finite(const char \*function, const T\_y &y, const char \*name, T\_result \*result)

Checks if the variable y is finite.

**Definition:** check\_finite.hpp:52

stan::prob::exponential\_rng

double exponential\_rng(const double beta, RNG &rng)

**Definition:** exponential.hpp:265

partials\_vari.hpp

stan::agrad::value\_of

T value\_of(const fvar< T > &v)

Return the value of the specified variable.

**Definition:** value\_of.hpp:16

constants.hpp

stan::agrad::OperandsAndPartials

A variable implementation that stores operands and derivatives with respect to the variable...

**Definition:** partials\_vari.hpp:76

traits.hpp

stan::return\_type::type

boost::math::tools::promote\_args< typename scalar\_type< T1 >::type, typename scalar\_type< T2 >::type, typename scalar\_type< T3 >::type, typename scalar\_type< T4 >::type, typename scalar\_type< T5 >::type, typename scalar\_type< T6 >::type >::type type

**Definition:** traits.hpp:406

stan::is\_constant\_struct

Metaprogram to determine if a type has a base scalar type that can be assigned to type double...

**Definition:** traits.hpp:57

stan::math::value\_of

double value\_of(const T x)

Return the value of the specified scalar argument converted to a double value.

**Definition:** value\_of.hpp:24

normal.hpp

boost::math::isinf

bool isinf(const stan::agrad::var &v)

Checks if the given number is infinite.

**Definition:** boost\_fpclassify.hpp:54

stan::prob::include\_summand

Template metaprogram to calculate whether a summand needs to be included in a proportional (log) prob...

**Definition:** traits.hpp:35

stan::math::SQRT\_2

const double SQRT\_2

The value of the square root of 2, .

**Definition:** constants.hpp:20

stan::prob::exp\_mod\_normal\_log

return\_type< T\_y, T\_loc, T\_scale, T\_inv\_scale >::type exp\_mod\_normal\_log(const T\_y &y, const T\_loc &mu, const T\_scale &sigma, const T\_inv\_scale &lambda)

**Definition:** exp\_mod\_normal.hpp:26

stan::agrad::sqrt

fvar< T > sqrt(const fvar< T > &x)

**Definition:** sqrt.hpp:15

stan::agrad::OperandsAndPartials::d\_x2

VectorView< double \*, is\_vector< T2 >::value, is\_constant\_struct< T2 >::value > d\_x2

**Definition:** partials\_vari.hpp:83

stan::prob::exp\_mod\_normal\_ccdf\_log

return\_type< T\_y, T\_loc, T\_scale, T\_inv\_scale >::type exp\_mod\_normal\_ccdf\_log(const T\_y &y, const T\_loc &mu, const T\_scale &sigma, const T\_inv\_scale &lambda)

**Definition:** exp\_mod\_normal.hpp:356

stan::math::check\_consistent\_sizes

bool check\_consistent\_sizes(const char \*function, const T1 &x1, const T2 &x2, const char \*name1, const char \*name2, T\_result \*result)

**Definition:** check\_consistent\_sizes.hpp:13

stan::prob::normal\_rng

double normal\_rng(const double mu, const double sigma, RNG &rng)

**Definition:** normal.hpp:377

stan::max\_size

size\_t max\_size(const T1 &x1, const T2 &x2)

**Definition:** traits.hpp:191

stan::math::check\_not\_nan

bool check\_not\_nan(const char \*function, const T\_y &y, const char \*name, T\_result \*result)

Checks if the variable y is nan.

**Definition:** check\_not\_nan.hpp:56

stan::agrad::OperandsAndPartials::d\_x4

VectorView< double \*, is\_vector< T4 >::value, is\_constant\_struct< T4 >::value > d\_x4

**Definition:** partials\_vari.hpp:85

exponential.hpp

value\_of.hpp

stan::agrad::OperandsAndPartials::d\_x1

VectorView< double \*, is\_vector< T1 >::value, is\_constant\_struct< T1 >::value > d\_x1

**Definition:** partials\_vari.hpp:82

stan::prob::exp\_mod\_normal\_cdf\_log

return\_type< T\_y, T\_loc, T\_scale, T\_inv\_scale >::type exp\_mod\_normal\_cdf\_log(const T\_y &y, const T\_loc &mu, const T\_scale &sigma, const T\_inv\_scale &lambda)

**Definition:** exp\_mod\_normal.hpp:252

stan::agrad::OperandsAndPartials::d\_x3

VectorView< double \*, is\_vector< T3 >::value, is\_constant\_struct< T3 >::value > d\_x3

**Definition:** partials\_vari.hpp:84

stan::math::pi

double pi()

Return the value of pi.

**Definition:** constants.hpp:77

stan::prob::exp\_mod\_normal\_rng

double exp\_mod\_normal\_rng(const double mu, const double sigma, const double lambda, RNG &rng)

**Definition:** exp\_mod\_normal.hpp:458

constants.hpp

stan::agrad::log

fvar< T > log(const fvar< T > &x)

**Definition:** log.hpp:15

stan::VectorView

VectorView is a template metaprogram that takes its argument and allows it to be used like a vector...

**Definition:** traits.hpp:275

stan::agrad::exp

fvar< T > exp(const fvar< T > &x)

**Definition:** exp.hpp:16

stan::math::negative\_infinity

double negative\_infinity()

Return negative infinity.

**Definition:** constants.hpp:123


---

[ Stan Home Page ]

*© 2011–2014,
Stan Development Team.*
